# An instrumented cylinder measuring pinch force and orientation

**DOI:** 10.1186/1743-0003-5-2

**Published:** 2008-01-02

**Authors:** Daniel Bourbonnais, Victor Frak, Jean-François Pilon, Michel Goyette

**Affiliations:** 1 Centre de recherche interdisciplinaire en réadaptation du Montréal métropolitain (CRIR), site Institut de réadaptation de Montréal, Montréal, QC, H3S 2J4, Canada; 2 École de réadaptation, Faculté de médecine, Université de Montréal, QC, H3C 3J7, Canada; 3 Département de kinanthropologie, Université du Québec à Montréal, QC, H3C 3P8, Canada

## Abstract

**Background:**

The function of a cylinder allowing simultaneous measurements of the opposition axis of the index finger and thumb of the hand and the magnitude of pinch force is described.

**Methods:**

The apparatus is made of two half-cylinders that are bonded together through a 6-axis force/torque sensor and allows the measurement of 3D orthogonal forces and moments of force. The amplitude of the pinch force exerted on the cylinder by the fingers is defined as the resultant of the forces in the different axes. A software program was developed to measure the barycentre of the forces on the instrumented cylinder, allowing calculation of the angle of the opposition axis between the fingers and the location of the resulting pinch force on the cylinder, assuming that the pinch or grip forces are co-linear through the center of the cylinder.

In order to assess the validity and reliability of the measurements, the cylinder was mounted on a milling table and seven calibrated weights (from 100 to 500 g) were successively applied perpendicularly to a 9*9 matrix of sites separated by 1 cm. With the exception of the extreme lateral parts of the cylinder, the dispersion of the calculated vertical position of the resulting force was always within 1 mm of the application point, suggesting a high reliability of these measurements. In addition, the errors in the angles of the applied force were calculated and found to be less than 2 degree with no clear patterns of variation across the different locations of the cylinder.

**Results:**

The usefulness of the cylinder is demonstrated by evaluating the pinch force and the opposition axis in six healthy subjects lifting the cylinder from the table using three different orientations of their right hand. The magnitude of the grip force was not significantly different across orientations (45, 22 and -22 degrees relative to the midline of the subject) suggesting that force grip is controlled.

**Conclusion:**

From these results, it has been concluded that the cylinder is a valid, reliable and precise instrument that may prove useful for evaluating opposition axis and grip force in healthy and pathological populations.

## Introduction

Grasping, holding and manipulating objects represent one of the most important functions of the hand. During the phase where the hand is brought into the vicinity of the object to be manipulated, the grasp aperture increases to reach a maximum before contact with the object and is adjusted precisely when the hand is close to the object [1]. In addition to controlling the aperture of the fingers, the orientation of the hand relative to the object is critical for effective manipulation. The grasp orientation as described by Napier [2] is determined from the configuration of the arm and the hand that the nervous system has to define in order to allow utilization of an object [3-5]. Moreover, the positions of the fingers on the object need to be determined by the nervous system to ensure secure manipulation. For example, pinching a cylinder requires the opposition of the index and the thumb to be approximately through the center of the object to ensure stability of the grip. The location, size and weight of the cylinders do not impact on the grasp orientation or the opposition axis, which remain stable with respect to an egocentric reference frame in right-handed individuals [6].

In addition to this spatial consideration, lifting and holding an object between the index finger and thumb requires fingertip shear forces to overcome the weight of the object and prevent it from dropping. The amplitude of the shear force is determined by the friction coefficient of the object and the amplitude of the grip force (GF, considered as the finger force acting perpendicularly to the object's surface). To avoid slipping and/or dropping of the object, the GF must therefore be modulated as a function of the friction coefficient and weight of the object [7,8]. Usually, subjects exert a slightly larger GF than the GF mechanically required to hold the object, providing a safety margin that allows small perturbations to be corrected without dropping the object [8]. Many studies have demonstrated the precise coordination between the GF and the shear forces during the manipulation of an object [8-11] but few have characterized their changes or magnitudes when the opposition axis or orientation of the hand is modified. Typically, the opposition axis is determined by the location of force transducers not allowing a person who lifts an object to select his/her preferred grasp orientation. This provides a rationale for developing an instrumented cylinder measuring both the amplitude of the pinch force and allowing self-selection or imposed orientation of the opposition axis. The precision of the measurements obtained from such an apparatus was investigated experimentally in the present study. Moreover, its usefulness is illustrated by exploring changes in GF across different configurations of the arm and hand while the individual is lifting the cylinder.

## Methods

### Experimental set-up

**Figure 1:**
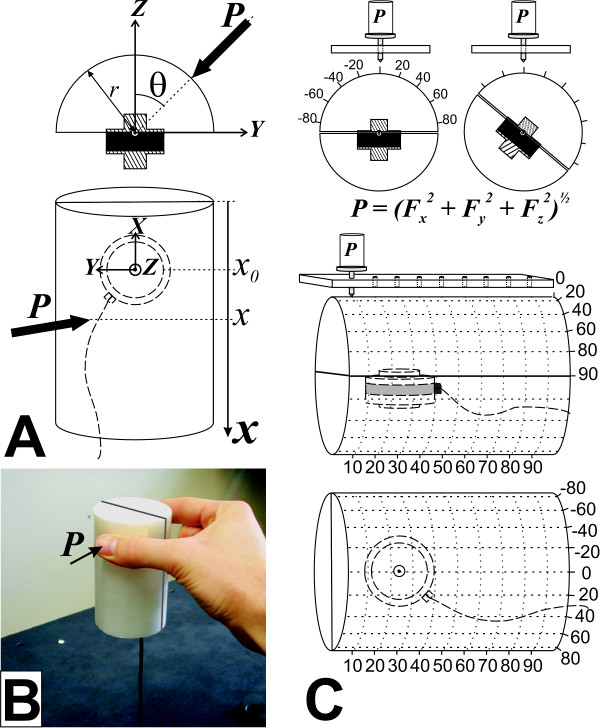
Functioning of the instrumented cylinder and experimental set-up used for its calibration. The apparatus is designed to measure the orientation (*θ*) and vertical location (*x*) of the applied force (*P*) by either the index or thumb while exerting a grip force (in A and B). These parameters are calculated from outputs of an F/T sensor (with axes *X*, *Y *and *Z*) embedded in the two half-cylinders using two T-adaptors (upper panel of A and C; T-adaptors indicated in hatched, the F/T sensor in black). In order to verify the accuracy of measurements, the cylinder was mounted on an angular positioning tool by clipping one of its ends, aligned horizontally, on a milling table (not illustrated) and we used a linear positioning plate (2 upper panels of C), in order to apply five different masses (*P *in 2 upper panels of C: 100, 200, 300, 400 and 500 g) vertically on each point of the matrices formed by the nine angles (-80° to +80°) and nine heights (10 to 90 mm) and indicated by the dashed lines. The sensitive side of the transducer is the one linked to the half-cylinder with only the T-adaptor inside it and not the body of the transducer (in black) itself (upper panels of A and C).

An instrumented cylinder (diameter of 60 mm and a height of 100 mm) having dimensional characteristics similar to everyday-life objects such as a glass or a bottle was built to allow the opposition axis and force magnitude to be measured while it is manipulated. The external frame of the cylinder consists of two separate nylon 66 half-cylinders rigidly connected to a single 6-axis force/torque (F/T) sensor (ATI Industrial Automation, NC, USA, model Mini 40 SI-20-1 with a resolution in each channel: *F*_*X*_, *F*_*Y *_= 1/100 N; *F*_*Z *_= 1/50 N; *T*_*X*_, *T*_*Y*_, *T*_*Z *_= 1/4000 Nm). All forces and moments of force exerted by the fingers are measured through the transducer since the two half-cylinders are separated by a gap of 0.56 mm. One of the half-cylinders is press-fitted and locked with set screws on an adapter that was fixed on the sensitive side ("*tool side*" as defined by ATI, see Figure 1C) of a single F/T sensor, enabling external forces and torques to be recorded (Figures 1A and 1C). The adapter consists of a machined hat-shaped nylon plate permitting adequate transfer of the forces and moments from the half-cylinders to the F/T sensor (Figures 1A and 1C, hatched area). The forces exerted on the non-sensitive half-cylinder are considered equal and opposite to the ones recorded on the opposite half-cylinder, assuming that stable manipulation of the instrumented cylinder is achieved.

In conjunction with the instrumented cylinder, a software program was developed (LabView v6.1, National Instruments, TX, USA) to record and process the 6-axis data from the F/T sensor. The software program provides a real-time display and recording of the experimental variables of interest, namely, opposition axis (*θ*), height (*x*) and resulting force (*P*) of the weight applied to the instrumented cylinder (Figure 1A). The center of pressure is expressed in terms of the angle and height of the force acting on the surface of the cylinder due to the applied loads (*θ *and *x *values in Figure 1A). All variables are obtained from computations based on the instrumented-cylinder characteristics (e.g. relative position of the F/T sensor, diameter) and measurements of the *XYZ *decomposed forces (*F*) and moments (*M*) captured from the F/T sensor embedded inside. The opposition axis was considered collinear through the cylinder's central axis. The program used the following equations to compute the variables of interest in a cylindrical coordinates referential (*r*, *θ*, and *x*, where *r *is fixed here to the radius of the cylinder and *x*_0 _is the position of the center of the F/T sensor relative to the top of the cylinder).

θ=arctan⁡(|FZFY|)−90
 MathType@MTEF@5@5@+=feaagaart1ev2aaatCvAUfKttLearuWrP9MDH5MBPbIqV92AaeXatLxBI9gBaebbnrfifHhDYfgasaacPC6xNi=xI8qiVKYPFjYdHaVhbbf9v8qqaqFr0xc9vqFj0dXdbba91qpepeI8k8fiI+fsY=rqGqVepae9pg0db9vqaiVgFr0xfr=xfr=xc9adbaqaaeGacaGaaiaabeqaaeqabiWaaaGcbaacciGae8hUdeNaeyypa0JagiyyaeMaeiOCaiNaei4yamMaeiiDaqNaeiyyaeMaeiOBa42aaeWaaeaadaabdaqcfayaamaalaaabaGaemOray0aaSbaaeaacqWGAbGwaeqaaaqaaiabdAeagnaaBaaabaGaemywaKfabeaaaaaakiaawEa7caGLiWoaaiaawIcacaGLPaaacqGHsislcqaI5aqocqaIWaamaaa@441A@

x=x0−cos⁡θ⋅r⋅FX−MYFZ
 MathType@MTEF@5@5@+=feaagaart1ev2aaatCvAUfKttLearuWrP9MDH5MBPbIqV92AaeXatLxBI9gBaebbnrfifHhDYfgasaacPC6xNi=xI8qiVKYPFjYdHaVhbbf9v8qqaqFr0xc9vqFj0dXdbba91qpepeI8k8fiI+fsY=rqGqVepae9pg0db9vqaiVgFr0xfr=xfr=xc9adbaqaaeGacaGaaiaabeqaaeqabiWaaaGcbaGaemiEaGNaeyypa0JaemiEaG3aaSbaaSqaaiabicdaWaqabaGccqGHsisljuaGdaWcaaqaaiGbcogaJjabc+gaVjabcohaZHGaciab=H7aXjabgwSixlabdkhaYjabgwSixlabdAeagnaaBaaabaGaemiwaGfabeaacqGHsislcqWGnbqtdaWgaaqaaiabdMfazbqabaaabaGaemOray0aaSbaaeaacqWGAbGwaeqaaaaaaaa@46F8@

P=FX2+FY2+FZ2
 MathType@MTEF@5@5@+=feaagaart1ev2aaatCvAUfKttLearuWrP9MDH5MBPbIqV92AaeXatLxBI9gBaebbnrfifHhDYfgasaacPC6xNi=xI8qiVKYPFjYdHaVhbbf9v8qqaqFr0xc9vqFj0dXdbba91qpepeI8k8fiI+fsY=rqGqVepae9pg0db9vqaiVgFr0xfr=xfr=xc9adbaqaaeGacaGaaiaabeqaaeqabiWaaaGcbaGaemiuaaLaeyypa0ZaaOaaaeaacqWGgbGrdaqhaaWcbaGaemiwaGfabaGaeGOmaidaaOGaey4kaSIaemOray0aa0baaSqaaiabdMfazbqaaiabikdaYaaakiabgUcaRiabdAeagnaaDaaaleaacqWGAbGwaeaacqaIYaGmaaaabeaaaaa@3A85@

#### Calibration

The instrumented cylinder was calibrated using precision devices including an angular positioning tool (milling table) and a linear positioning plate (Figure 1C). The cylinder was firmly clipped horizontally on the angular positioning tool in order to be able to rotate it and to measure the weights vertically applied to its surface. The angular positioning tool had a vernier, giving a measurement precision of 0.1°, while the positioning plate comprised circular, center-spaced holes of 1 cm in which the weight support could be inserted. The weight support had a pointed tip allowing the standard weights to be placed precisely (combination of angle and height) on the surface of the cylinder. Also, the weight of this support was subtracted from the load measured by the cylinder, since a baseline was established before recording began, while the weight support was in contact with the surface. With the instrumented cylinder weighing approximately 100 g, the range of weights used for the calibration was dictated by the most probable forces exerted by a subject manipulating the object. Multiple combinations of standard weights (100, 200, 300, 400 and 500 g) and positions (angles ranging from -80° to 80° in increments of 20°, with 0° being aligned with the center of the force cell flat surfaces; height ranging from 10 to 90 mm in increments of 10 mm from the top of the cylinder; see Figures 1A and 1C) were used for the purpose of calibration (5 weights × 9 angles × 9 heights = 405 combinations). The opposition axis (*θ*), height of precision grip (*x*) and resulting force (*P*) were averaged for each combination from 500 data points acquired during a 5-s acquisition period at 100 Hz.

### Empirical data processing for calibration

A descriptive statistical analysis including computation of means, standard deviations (SD), confidence intervals at 95% (CI) and absolute error (AE) of each *P *value obtained was performed on the calibration data recorded when applying a given weight to the cylinder at the different locations (*x *and *θ*). For the center of pressure (COP) variables (*θ*, *x*), the mean, SD, CI and AE values were computed by averaging data for the different weights applied to the cylinder for each combination of *θ *and *x *(matrix of 9 × 9, see Figure 1C).

The weights used to determine the amplitude of *P *were precise (1000 g × 10 g Brass Hook Weight Set, item# 46206-00, Ohaus, USA) and were used as gold standards. In contrast, the experimental set-up had its own sources of error regarding the location of the applied force, as will be addressed later in the discussion. Thus, for the COP variables, the theoretical values of *θ *or *x *chosen in the spatial referential determined by the experimental set-up (see Figure 1C) were considered as absolute while their averaged values over all the weights and for all *θ *or *x *(depending on the variable of interest) were used to obtain gold standards. Before obtaining gold standards, we intended to minimize the difference between all points from the absolute referential and the averaged values computed for all *θ *or all *x *in order to detect a possible systematic error introduced in the measurements by the experimental set-up. The AEs difference between expected theoretical value and averaged empirical value) on the COP variables indicated a tendency in the measurements to systematically over- or undershoot the expected measures. Concretely, the measurements for the angles tested were averaged for all weights and heights tested at a single angle and vice-versa for the heights tested. This procedure revealed a systematic offset of about 1.5° and 0.5 mm between the theoretical referential and the computed means of angle and height measurements, respectively. This offset was always over (heights) or under (angles) the expected values, representing a systematic error in the empirical data that we subtracted from all the empirical measurements, i.e. before obtaining the COP gold standards. The AEs on the COP variables were then computed as the difference between the mean of the variable of interest over all the weights at a specific point (*θ*, *x*) and the gold standards (both corrected for systematic offset), while the AEs on weights were simply the difference between the measured and standard weights used during calibration.

For *P *statistics, the use of standard weights (gold standards) allowed us to compare each measurement with a specific weight (9 heights × 9 orientations = 81 combinations) to the real weight applied. On the other hand, to determine the spatial precision and accuracy of the instrumented cylinder measurements, we relied on means, SDs and CIs given by the frequency distribution of the COP variables. Means and SDs allowed us to verify that the distributions of COP variables were centered near the expected values (based on the calibration set-up referential) and presented a narrow deviation from the average, which produced a high precision and accuracy. CIs gave us another way of visualizing the precision of the instrument, since 95% of the measurements performed lie within the range of the CI.

### Variations of pinch force using different orientations

**Figure 2:**
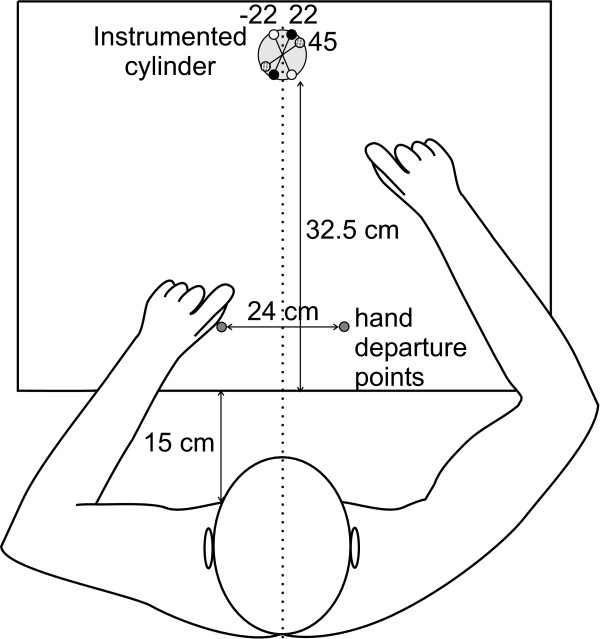
Experimental set-up for testing grasps orientations. A subject is required to execute successive series of lifts of the instrumented cylinder to a height of approximately 10 cm using his right hand with the finger pads aligned at different orientations identified by colour markers on the top of the cylinder (dashed: 45°, black: 22° and white: -22°).

Six subjects (two men and four women aged between 21 and 45 years old) participated in the study. This experiment was approved by the research ethics committee of our institution and all participants gave their informed consent before the study began. All subjects were right-handed, as evaluated using the 'Edinburgh Handedness Inventory' (Oldfield, 1971). The subject sat on a chair without armrests facing the instrumented cylinder placed at his/her midline on a table. His/her trunk was located 15 cm from the edge of the table, 32.5 cm from the cylinder, which was placed on the table (Figure 2). The subject's hands were placed on the table, 12 cm each side of the midline. At the request of the experimenter, the subject was asked to reach, pinch and lift the cylinder at his/her own pace with the right thumb and index finger pads to a height of approximately 10 cm and maintain this position for 10 s (Figures 1B and 2). The finger pads had to be aligned with one pair of colour markers (red: 45°, green: 22° and yellow: -22°) visible on the top of the instrument and indicating the approximate opposition axis required. Only visual inspection by the experimenter was used to determine whether subjects had used the opposition axis required and the exact opposition axis was calculated using the instrumented cylinder. It is important to note that the reference axis for the angle measurements is the *Z *axis of the transducer, as shown in Figure 1A. Therefore, each marker pair was positioned on the top extremities at clockwise angles relative to the subject's medial line (see Figure 2). Ten trials were performed for each of the three different orientations (45°, 22° and -22°). The wrist joint amplitude was measured with an electro-goniometer (model SG65, Biometrics Ltd., UK). Each condition was evaluated 10 times and the order was randomly administered for each subject. The amplitude of the force applied by the finger pads (*P*), their opposition axis (*θ*) and their height (*x*) relative to the top of the cylinder were given by the instrumented cylinder. The movement was divided into two main phases: a dynamic phase, when the object is lifted from the table, and a static phase, when the object is stabilized in the air before being deposited on the table. These two phases were chosen because the grip forces observed during the dynamic phase were noticeably different from those measured during the static phase due to the inertial forces caused by acceleration and deceleration of the object and the following adaptation of grip force while maintaining the object in the air [10,12]. The beginning of the dynamic phase corresponded with the onset of the GF and was determined as the first point exceeding 2 SD from the mean baseline of GF and having a positive derivative of GF (*d*GF) for at least six consecutive points. The end of the dynamic phase was temporally defined as 1450 ms after the first minimal amplitude of *d*GF following the maximal amplitude of GF. This period of 1450 ms was arbitrarily chosen after visualizing numerous empirical signals. We analyzed the data for two selected periods of 250 ms measured respectively while the subject lifted the object in the air (maximal amplitude of the dynamic period) and about 5 s after the subject had been holding the object in the air (middle of the static period). The pinch force value and the parameters of the axis of opposition were then averaged in these two windows.

## Results

### Angle, height and weight (force) calibration measurements

**Figure 3:**
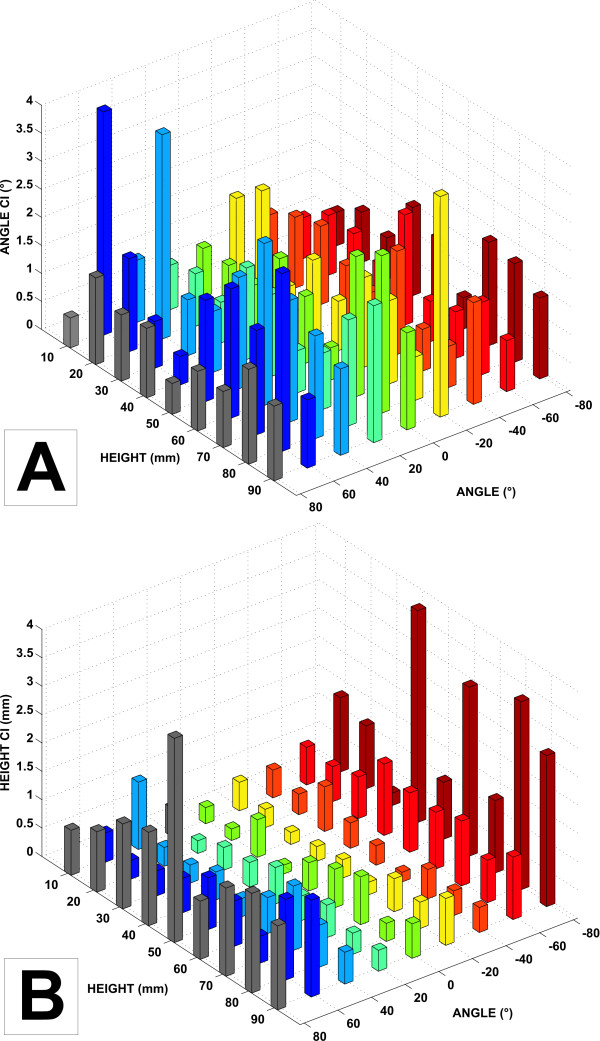
A) Distribution of the 95% confidence intervals for empirical angles recorded during the calibration of the instrumented cylinder. Results are averaged for all the weights. B) Same confidence intervals but for calculated heights.

**Figure 4:**
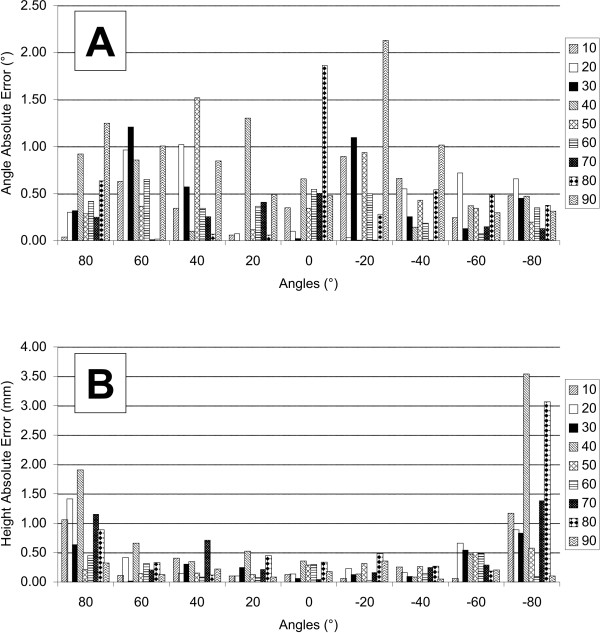
A) Distribution of the absolute error between the angles computed by the instrumented cylinder and the expected angles (absolute referential) at all angles and heights. B) Same absolute errors but for heights.

As presented in Figures 3A, 4A and Table 1, no clear pattern could be identified in the empirical errors calculated relative to the mean of the experimental angles, using the five different weights at the different locations of the cylinder surface. The empirical errors are shown for all angles (abscissa) and all heights (*x *axis) tested (see legend). No clear patterns were observed across the different angles and heights tested. The angular mean AE and mean variance for all the locations on the cylinder are respectively 0.48° and 1.65° (mean SD = 1.13°).

**Table 1:** Angle measurement precision evaluated by different indicators based on absolute (mean and standard deviation – SD), root mean square (RMS) and maximum (max.) errors.

***Theoretical***									
**Angle Tested**	**80.00**	**60.00**	**40.00**	**20.00**	**0.00**	**-20.00**	**-40.00**	**-60.00**	**-80.00**
***Empirical Angles***									
Mean* (N)	80.47	59.48	40.14	20.13	-0.25	-20.237	-40.05	-60.00	-79.66
**Mean SD (N)**	0.91	1.55	1.54	1.04	1.15	1.255	0.95	0.84	0.96
Mean Error** (N)	0.49	0.64	0.56	0.32	0.54	0.654	0.42	0.31	0.38
**SD Error (N)**	0.38	0.43	0.48	0.41	0.54	0.699	0.31	0.20	0.16
**RMS Error (N)**	0.61	0.75	0.72	0.50	0.74	0.928	0.51	0.37	0.41
**Max Error (N)**	1.25	1.21	1.52	1.30	1.87	2.130	1.02	0.72	0.66

* Corrected for systematic offset of -1.51°.

** Relative to the corrected empirical mean

The amplitude of the errors for the experimental heights was higher at the extreme angles and at lower heights (Figures 3B, 4B and Table 2) of the half-cylinder, resulting in an asymmetrical bowl-shaped distribution. The mean AE and mean variance of the heights were 0.43 mm and 0.83 mm respectively (mean SD = 0.66 mm).

**Table 2:** Height measurement precision evaluated by different indicators based on absolute (mean and standard deviation – SD), root mean square (RMS) and maximum (max.) errors.

***Theoretical***									
**Heights Tested**	**10.00**	**20.00**	**30.00**	**40.00**	**50.00**	**60.00**	**70.00**	**80.00**	**90.00**
***Empirical Heights***									
Mean* (mm)	10.19	20.01	30.18	40.15	50.12	59.77	69.74	80.01	89.71
**Mean SD (mm)**	0.56	0.41	0.47	0.81	0.78	0.71	0.72	0.86	0.87
Mean Error** (mm)	0.37	0.46	0.32	0.89	0.29	0.22	0.49	0.68	0.18
**SD Error (mm)**	0.44	0.45	0.29	1.13	0.16	0.17	0.48	0.92	0.11
**RMS Error (mm)**	0.55	0.63	0.42	1.39	0.32	0.27	0.67	1.10	0.21
**Max Error (mm)**	1.17	1.42	0.83	3.54	0.58	0.49	1.38	3.06	0.36

* Corrected for systematic offset of 0.51 mm

** Relative to the corrected empirical mean

Finally, the magnitudes of the applied forces (weights) were calculated for all locations tested and the mean values and the errors are presented in Table 3. The difference between the applied force and the measured force by the transducer was less than 0.165 N for the range applied (0.19 N to 4.9 N). The mean AE ranged from 0.014 N to 0.034 N for all the weights used while the coefficients of variations ranged from 0.006 to 0.049.

**Table 3:** Weight measurement precision evaluated by different indicators based on absolute (mean and standard deviation – SD), root mean square (RMS) and maximum (max.) errors.

***Theoretical***					
**Mass tested (g)**	**100**	**200**	**300**	**400**	**500**
**Weight (N)**	0.981	1.961	2.942	3.922	4.903
***Empirical Weights***					
**Mean (N)**	0.991	1.982	2.968	3.948	4.924
**SD (N)**	0.014	0.015	0.025	0.030	0.031
**Mean Error (N)**	0.014	0.022	0.032	0.034	0.029
**SD Error (N)**	0.009	0.013	0.017	0.021	0.025
**RMS Error (N)**	0.017	0.026	0.036	0.039	0.038
**Max Error (N)**	0.035	0.057	0.088	0.082	0.165

### Pinch force across opposition axes

**Figure 5:**
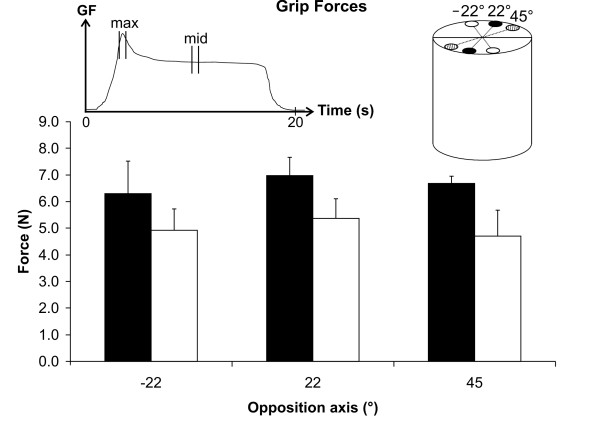
Mean and standard deviation values of the right hand grip forces estimated during the dynamic (max – black) and static (mid – white) phases (left inset) of movement for the three axes of opposition tested (-22°, 22° and 45°, right inset).

The amplitude of the forces (*P*) measured during the dynamic (max) and static (mid) phases of the manipulation of the cylinder for the different opposition axes is illustrated in Figure 5 and summarized in Table 4. The ANOVAs indicate that the forces (*P*) exerted did not differ across the different opposition axes for both the dynamic (*F*(2,10) = 0.538 *p *= 0.573) and static phases (*F*(2,10) = 1.03 *p *= 0.369).

**Table 4:** Results of the experimental pinch and lift of the cylinder with right-hand fingers at three different opposition axes

	***Dynamic phase***			***Static phase***		
**Orientations required (°)**	-22	22	45	-22	22	45
**Grip force (N)**	6.30 (1.22)	6.98 (0.69)	6.68 (0.27)	4.92 (0.80)	5.37 (0.74)	4.71 (0.97)

**Axis of opposition measured (°)**	-21.38 (1.87)	29.01 (1.67)	50.96 (1.30)	-20.50 (1.55)	29.39 (2.36)	50.99 (2.15)
**Heights (cm)**	5.12 (0.11)	4.97 (0.21)	4.89 (0.19)	5.50 (0.17)	5.33 (0.28)	5.19 (0.12)
**Wrist angle (°)**	-27.21 (7.34)	20.94 (4.41)	35.67 (4.82)	-23.19 (5.80)	19.78 (3.91)	33.91 (5.17)

The mean axis of opposition measured by the apparatus during the dynamic and static phases was significantly different from the required angles for both the 22° and 45° orientations (t values varying between 6.82 and 11.25 and p value situated between 0.001 and 0.0001) but not for the -22° orientation (t values -0.810 and 2.36 with a p value of 0.455 and 0.064 respectively for the dynamic and static phases). This difference between the required and measured orientation is probably associated with visuo-motor mechanisms that caused uncertainties in the spatial positioning of the fingers on the object.

The mean heights were significantly different across orientations for both the dynamic (F(2,10) = 4.55 p = 0.047) and static phases (F(2,10) = 11.67 p < 0.02). Contrast analyses indicated that the height in the direction of -22° differs from 22° and 45° for both the dynamic (t = 2.80; p = 0.038; t = 2.76; p = 0.04) and static phases (t = 2.80 p = 0.038; t = 5.83 p = 0.002).

The joint amplitude of the wrist was also measured at the dynamic and static phases and differs for the different opposition axes. The ANOVAs indicate that the joint amplitude measured is different across the different orientations for both the dynamic (*F*(2,10) = 188.28 *p *< 0.001) and static phases (*F*(2,10) = 177.36 *p *< 0.001). Post-hoc analyses indicate that the joint amplitudes differ between each of the different opposition axes for both the dynamic and static phases.

## Discussion

The functioning of a cylinder allowing the axis of opposition and the magnitude of the force applied between the index finger and thumb to be measured during a pinch is described. Experimental errors in measurements were evaluated and the application of the apparatus was demonstrated by measuring the pinching forces at three different orientations in healthy subjects.

In order to calibrate the cylinder, weights were applied vertically through a positioning plate at different locations on the surface of the cylinder. The different locations were tested by rotating the cylinder (angles) using a milling table and using different holes of the position plate (heights). This experimental set-up generated systematic and random errors, thus affecting the location and the magnitude of the applied force. One example of such a systematic error, given in the Results section, was probably due mainly to the initial position of the object in the calibration set-up, which was evaluated visually using a ruler. In addition, the fitting of the force/torque sensor inside the cylinder was slightly imperfect, potentially contributing to the systematic error in the torque and force measurements due to an incorrect alignment of the sensor with respect to the cylinder. However, a procedure was implemented to take into account the systematic errors occurring due to misalignment of either the applied force and/or the position of the cylinder under the positioning plate set using the milling table. Systematic errors of 1.51 mm and 0.51° were calculated and removed from the averaged values of COP variables in order to obtain gold standards as close as possible to the theoretical values. In addition, random errors in the angle or height measurements may result from slight displacements of the weight support within the guide system and also from errors occurring while positioning the cylinder using the milling table. The slight misalignments of the applied force with respect to the referential coordinates of the force transducer may generate an error in the magnitude of force, since the force is exerted at a slight angle. Nonetheless, assuming that this angle value is small, these errors were estimated to be very low, since they vary as a cosine function. That being said, the slight misalignment of the weight support inserted into the guide hole of the positioning plate and the uncertainties in the positioning of the cylinder under the position plate using the milling table can also introduce an error in the location of the applied force, generating a source of error in the angle and height measurements. We estimated *visually *that the maximal random error in the positioning of the applied force on the cylinder is approximately 0.5 mm, which is close to the mean AE calculated for heights (Figure 4B). Since the cylinder has a radius of 3.0 cm, we calculated that an error of location of 0.5 mm would correspond to an angular error of approximately 1°, which is also close to the AEs that were estimated (Figure 4A). Other random sources of error could be due to the mechanical assembling of the two separate half-cylinders. These parts were slightly separated and the compliance of the material could introduce a change in the radius of the cylinder while applying forces on it. However, it was carefully verified that no contact between the two halves of the cylinder occurred within the range of force tested. Finally, the minimal resolution of the force/torque sensor, which is of the order of 1/50 N for force and 1/4000 Nm, also contributes to the random error of measurement. In sum, although different sources of error could affect the validity of height and angular measurements, their empirical estimations were found to be rather small (less than 0.5 mm and less than 1.0°).

The results indicate no clear pattern or difference in the AEs of angular measurements across the surface of the cylinder. In contrast, the AEs in the height measurements tend to increase as the force application is displaced laterally (towards +80° or -80°) or, to a lesser degree, distally (towards 90 mm). This height is defined in the *X *coordinates of the sensor (Figure 1A) and estimated by the ratio of torques and force components in the *Z *axis. In contrast, the angular measurements are estimated directly from a ratio of the force values. The relative contribution of the moments of force with respect to the *F*_*z *_component of force probably introduces the errors shown. In this regard, an important limitation of the design of the apparatus is that the length and width of the surface of the cylinder exceed those of the sensor, implying that forces and moments of force are also applied outside the surface of the sensor. It is probable that this increase in measurement errors as the forces are applied laterally and distally could be minimized by using two force sensors at each end of the cylinder or by improving the design of the interface between the sensor and the cylinder.

Although the apparatus developed has some limitations, it offers great potential for measuring the opposition axes of the fingers compared to other methods. The spatial and kinetic precision achieved is expected to be superior to a movement analysis system using position sensors. With position sensors on the tips of the index finger and thumb, the estimation of the axis of opposition would be prone to error, since the surface of contact between the skin and cylinder is quite large so the force could be applied at different locations and orientations, considering that the musculoskeletal apparatus and even the skin of the fingertips are deformable. A more precise evaluation of the opposition axis and height of the force applied on an object are therefore obtained by computing spatial coordinates from force and torque recordings. However, calculation of the axis of opposition has one limitation, that the real opposition axis of the fingers is presumed to cross the center of the cylinder. Should the fingers not be aligned with the center of the cylinder, the opposition axis calculated and identified by the software would be slightly lacking in precision compared to the real one.

Several studies [6,13,14] have indicated that the natural axis of opposition used by right-handers when manipulating a cylinder is 35° on average, ranging from 0° to 68°. The present results indicate that, when manipulating the cylinder using their right hand, right-handers generated an equivalent level of GF at opposition axes of 45°, 22° and -22°. The force levels were observed to be similar across orientations for both the dynamic and static phases of manipulation of the object. Therefore, although the different subjects had interacted using different sequences of orientations with the object, the forces were almost identical in all three orientations. These results suggest that the coordination between the grip and load force is maintained independently from the axis of opposition on the object. This opens up interesting avenues of research, since the axis of opposition has rarely been a controlled variable in the vast literature examining neurological mechanisms involved in the kinematic control of GF. Indeed, this indicates that the grip force is kept constant even though the geometrical configuration of the arm and hand are modified and muscles lengths are changed. It is therefore suggested that a central mechanism helps to control the grip force.

## Conclusion

A novel instrumented cylinder allowing simultaneous measurement of the opposition axis (angle, height) of the fingers and the GF they produce when lifting it has been presented. This cylinder has been calibrated and the results show great precision and accuracy of the measurements (angle about 1° and height about 0.5 mm on average for absolute error). A simple experiment showed that forces and opposition axis can be measured by means of this novel instrument. No significant differences between force amplitudes were observed for the different orientations (opposition axis) for both the dynamic and the static period of grip. We propose this tool as a precision measuring instrument for the assessment of various everyday pinch tasks in the context of behavioral studies.

## Competing interests

The author(s) declare that they have no competing interests.

## Authors' contributions

DB designed the mechanical set-up and supervised the development and construction of the instrumented cylinder; conceptualized the method of calculation of the axis of opposition; contributed to the development of the experimental protocol and analysis of the data related to the sources of measurement of the cylinder and axes of opposition in human subjects, and was involved in the writing of the manuscript. VF provided the impetus to develop the apparatus by identifying the need; contributed to the design of the experimental protocols and analysis of the data related to both the sources of error of the cylinder measurements and the axes of opposition in human subjects, and was involved in the writing of the manuscript. JFP was involved in the analysis and interpretation of the data regarding the source of errors in the cylinder measurements; was involved in the writing of the manuscript and produced the figures; analyzed the data and helped to interpret the data obtained in human subjects. MG contributed to the development of the methodology used to calculate the variables using force plate inputs, and developed and implemented the software used in the study. All authors have read and approved the present manuscript.
